# Updates in Neurocritical Care

**DOI:** 10.1155/2018/1617359

**Published:** 2018-08-01

**Authors:** Christos Lazaridis, Laith Altaweel, Dimitrios Karakitsos

**Affiliations:** ^1^Division of Neurocritical Care, Departments of Neurology and Neurosurgery, University of Chicago, Chicago, IL, USA; ^2^Department of Medicine, Inova Fairfax Hospital, Falls Church, VA 22042, USA; ^3^Department of Critical Care, Keck Medical School, USC, Los Angeles, CA, USA; ^4^Critical Care Department, King Saud Medical City, Riyadh, Saudi Arabia

Caring for acutely and critically ill neurological and neurosurgical patients comprises the focus of the specialty of neurocritical care. It should be recognized that in reality, the care of such patients requires the expertise and involvement of a number of specialists including intensivists, neurologists, neurosurgeons, anesthesiologists, and specialized nurses. Although it is not only about “saving brain,” admittedly successful neurocritical care is care that salvages injured brain tissue and enhances the prospects of neurologic recovery. Hereby, we briefly mention recent clinical updates in neurocritical care; several of them are further and thoroughly discussed in the manuscript collection of this volume. The areas to be selectively highlighted in this short piece include the following: (a) management of large vessel occlusion (LVO) ischemic stroke, (b) global strategies for brain monitoring and protection after cardiac arrest and during extracorporeal cardiorespiratory support, (c) the approach towards monitoring and managing severe traumatic brain injury, and (d) minimally invasive neurosurgical approaches for intracerebral and ventricular hemorrhages.

Until recently, intravenous tPA was the only evidence-based treatment for acute ischemic stroke. More recently however, a number of high-quality multinational randomized controlled trials (RCTs) have proven that for selected patients presenting with LVO up to 24 hours from symptom onset, endovascular recanalization improves functional neurologic outcome, when compared to medical therapy. Six RCTs have independently demonstrated clinical benefit of stent-retriever thrombectomy when performed within 6 hours from stroke onset (REVASCAT, SWIFT PRIME, EXTEND-IA, ESCAPE, THRACE, and MR CLEAN). THRACE and MR CLEAN independently demonstrated benefit based solely on noncontrast CT and demonstration of LVO; consequently, these are the recommended imaging eligibility criteria. Two other trials have provided evidence for extending the window even further based on additional perfusion imaging and demonstration of a favorable ratio between salvageable brain and infarcted core (DEFUSE 3: 6–16 hours; DAWN: 6–24 hours) [1]. Based on the aforementioned literature, there is a clear imperative evaluating patients presenting with an NIHSS >5 for LVO, and depending on timing size of ischemic infarct core, and penumbra. Challenges that remain relate to the availability of experts and organization of care towards centers that can offer expedited, specialized clinical and radiographic assessment followed by prompt intervention for eligible patients. Additional questions for future research include extending the time window further, or abandoning all together “time from onset” as an eligibility criterion and replacing it with imaging eligibility criteria.

Outcomes of patients who survive cardiac arrest are critically dependent on the degree of global brain injury sustained. The earlier days of enthusiastic endorsement of therapeutic hypothermia with the goal of 32–34°C, based on the 2002 RCTs by Bernard et al. and the HACA study group, have been tempered by the more recent, and larger, Nielsen et al.'s study [2–4]. The latter study found that 33 versus 36°C were equivalent targets in terms of mortality and neurologic outcomes for patients with out-of-hospital cardiac arrest. A detailed comparison of these studies is out of scope, but it is worth noting that the Nielsen study had a remarkably high rate of expedient bystander application of CPR (an unrealistic situation in most parts outside of Scandinavia) and that what Nielsen et al. compared were two different strategies of targeted temperature management (TTM) rather than what the earlier RCTs tested, mild hypothermia versus no temperature control. As a result, there is ongoing debate on what the most appropriate target may be within the 32–36°C, and if there are patient characteristics or monitoring modalities (or biomarkers) that could provide patient-specific targets. Nevertheless, what should be uncontroversial is that TTM is the indicated brain protective strategy for 24–48 hours after cardiac arrest and that temperatures above 36–37°C are to be prevented with maintenance of normothermia thereafter. Global anoxic-ischemic brain injury in addition to more focal ischemic and hemorrhagic strokes has been observed, in nontrivial rates, in patients subjected to extracorporeal membrane oxygenation (ECMO). In view of the increasing use of ECMO modalities for the management of various causes of respiratory and/or cardiac failure, it is important that we consider methods to prevent, monitor, and potentially ameliorate brain injury in this setting. Unfortunately, current tools for noninvasive monitoring of cerebral blood flow (CBF), brain oxygenation, and energy dynamics are not advanced enough to be informative in optimizing patient-specific brain physiology. More study is clearly required while use of transcranial Doppler (TCD) and near-infrared spectroscopy (NIRS) has been reported with interest. Interestingly, the ability to monitor the status of CBF autoregulation after cardiac arrest and during ECMO may shed light to pathophysiologic mechanisms.

The value of invasive, multimodality monitoring is another passionately debated topic when it comes to managing patients with severe traumatic brain injury. It seems that there are two camps of thought; one sees great potential in obtaining compartmental pressures, tissue oxygenation, and metabolic data with a goal of tailoring interventions to the individual patient and according to physiologic principles of oxygen delivery, demand, and utilization. The second camp maintains skepticism towards the aforementioned approach arguing on the basis of a lack of hard data for improved patient outcomes in the face of potential adverse effects, resource utilization, and cost. This debate has not been helped by recent RCTs addressing specific interventions against refractory intracranial hypertension. The Eurotherm 32-35 trial established that hypothermia should not be used early (i.e., before other stage 2 treatments such as osmotherapy) in patients with diffuse TBI, despite beneficial effects on intracranial pressure (ICP) control [5]. The clinical outcomes of patients in the intervention arm of this study were worse than those of control patients. More recently, the RESCUEicp trial sought to establish the role of decompressive craniectomy (DC) for treating refractory ICP [6]. This trial took about 10 years to complete, involving 52 centers in 20 countries to recruit 408 patients. While there was a substantial mortality benefit with surgical decompression (for every 100 patients treated surgically versus medically there were 22 more survivors), it came at the expense of increased rates of moderate and severe disability when compared to the medical management cohort. Such results require careful, engaged, and transparent conversations with families during shared decision-making to avoid cognitive biases that may derive goals of care towards either an overly pessimistic or nihilistic stance or, on the contrary, be influenced by unrealistic optimistic biases. Finally, a promising step was made in favor of the monitoring camp by the completion of the BOOST phase II trial demonstrating that adding brain tissue oxygenation (PbtO_2_) monitoring to standard ICP/CPP resulted to less cerebral hypoxia [7]. The forthcoming phase III trial will determine whether PbtO2-targeted therapy will improve long-term neurologic outcomes.

Ischemic stroke, traumatic brain injury, neuromonitoring, and global cerebral protection are among the conditions that the field can claim some developments and successes. The same cannot be said for nontraumatic, non-coagulopathy-related intracerebral and/or intraventricular hemorrhages (ICH/IVH). These types of hemorrhages remain the most debilitating type of stroke with no specific treatments that can alter their course for the better. The promise of finding the optimal blood pressure target remains elusive with more aggressive control to possibly confer modest benefits in terms of hematoma expansion but no proof that it improves patient outcomes. Surgery trials have also failed in this regard with the possible exception of superficial lobar hemorrhage cases. Hope has been placed on minimally invasive approaches where a less morbid way of evacuating intraparenchymal or clearing intraventricular blood may eventually confer either clinical outcome benefits or secondary benefits such as faster resolution of hydrocephalus or a decreased need for shunt placement. The relevant studies are the recently completed CLEAR IVH III and the ongoing MISTIE-III and ENRICH trials. CLEAR-III tested patients with ICH <30 cc and large IVH causing hydrocephalus for improved clinical outcomes when given low-dose rtPA via an external ventricular drain versus placebo. Despite a reduction in mortality, no functional outcome benefit was seen in the intervention arm [8]. MISTIE-III seeks to demonstrate that minimally invasive surgery (MIS) plus rtPA for intraparenchymal hematoma evacuation, and for three days, improves functional outcome by a 12% increase in the modified Rankin Scale (mRS) score 0–3 compared to medically treated subjects assessed at 180 days; it is underway involving 90 centers in the US, Europe, Israel, China, and Australia (ClinicalTrials.gov Identifier: NCT01827046). Finally, ENRICH is a multicenter, randomized, adaptive clinical trial comparing standard medical management to early surgical hematoma evacuation (less than 24 hours) using minimally invasive parafascicular surgery (MIPS) in the treatment of intracerebral hemorrhage (ClinicalTrials.gov Identifier: NCT02880878).

The science and clinical medicine of “saving brain” are still at an early stage. Notwithstanding, there have recently been significant developments for a number of conditions. We have highlighted a few of these without an intention to be exhaustive; the manuscripts in this special issue on updates in neurocritical care cover some of these and other relevant topics in depth.

*Christos Lazaridis* *Laith Altaweel* *Dimitrios Karakitsos*
